# Impact of Canadian Wildfire-Emitted Particulate Matter on THP‑1 Lung Macrophage Health and Function

**DOI:** 10.1021/acs.est.4c10304

**Published:** 2025-02-18

**Authors:** Lila Bazina, Glen Deloid, Luke Fritzky, Denisa Lizonova, Nachiket Vaze, Philip Demokritou

**Affiliations:** † Nanoscience and Advanced Materials Center, Environmental and Occupational Health Sciences Institute (EOHSI), 40547 Rutgers University, Piscataway, New Jersey 08854, United States; ‡ Department of Environmental Occupational Health and Justice, School of Public Health, Rutgers University, Piscataway, New Jersey 08854, United States; § New Jersey Medical School, Cancer Institute of New Jersey, Rutgers University, Newark, New Jersey 07103, United States

**Keywords:** Canadian wildfires, wildfire particulate matter, THP-1 macrophages, climate change

## Abstract

Increasing frequency and intensity of climate-driven wildfires in recent years have resulted in increased human exposures to wildfire smoke and raised serious public health concerns. One potential risk of wildfire smoke exposure is the impairment that it may cause to lung macrophages, which serve as the first line of defense against inhaled pathogens and particles. Size-fractionated wildfire particulate matter (WFPM) collected in the New Jersey/New York metropolitan area during the June 2023 Canadian wildfire event was used to assess the effect on the health and function of THP-1 lung macrophages. Environmentally relevant in vitro WFPM doses were determined using established in vivo and in vitro dosimetry models. Exposure to WFPM_0.1–2.5_ (0.1–2.5 μm) for 24 h caused a significant (∼15%) increase in reactive oxygen species, indicating oxidative stress. More importantly, exposure to either WFPM_0.1_ (≤0.1 μm) or WFPM_0.1–2.5_ significantly reduced THP-1 lung macrophage viability. Additionally, 24 h exposure to either of the WFPM fractions reduced phagocytosis of unopsonized 1 μm polystyrene beads by approximately 50%, which appeared to be due to a defect in binding, which could in turn be a result of scavenger receptor blockade by WFPM or diminished viability and thus ATP depletion, depriving the macrophages of energy required to perform phagocytosis. Together, these findings suggest that WFPM exposure could impair lung macrophage health and function, which could increase susceptibility to respiratory infections. Further mechanistic in vitro and in vivo studies are warranted to better understand the impacts of WFPM on lung innate immunity and the risk of pulmonary infection.

## Graphical abstract

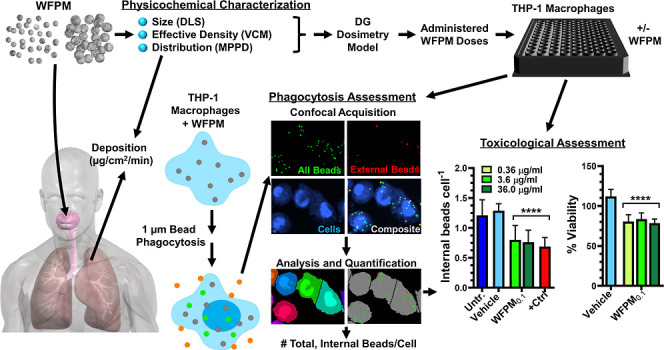

## 1 Introduction

Wildfires are significant environmental events that occur globally, affecting various ecosystems and communities^1,2^ and having extensive environmental and public health implications.^3,4^ The rising frequency and intensity of wildfires are primarily attributed to climate change, which elevates global temperatures and exacerbates drought conditions.^5–7^ As temperatures increase, so does the likelihood of severe weather events, such as prolonged droughts and changes in precipitation patterns, which create favorable conditions for wildfires.^8^ In addition, the overgrowth of forests in the U.S. over the last century has amplified the effects of climate change by providing an overabundance of kindling and fuel that facilitate the initiation and growth of wildfires.^9^

It is anticipated that the number and intensity of wildfires will increase by 50% by 2100.^10^ Over the past four decades, the annual average of forested land destroyed by wildfires in the United States has surged by 1000%.^11^ Each year, roughly 60,000 wildfires in the United States consume an average of 7 million acres of land.^12^ The duration of wildfire seasons in recent years has also increased significantly.^13^ In 2020, more than 28 million residents of California (70% of the state’s population) endured over 100 days of poor air quality due to wildfire smoke, characterized by elevated levels of particulate matter with a diameter ≤2.5 μm (PM_2.5_).^14^ During the 2020 wildfires in California, daily concentrations of PM_2.5_ often reached 350–500 μg/m^3^, which greatly exceeded the 24 h average limit of 35 μg/m^3^ specified by the National Ambient Air Quality Standards (NAAQS).^15^ In addition, 15 U.S. states have reported experiencing one or more days of PM_2.5_ concentrations exceeding the US Environmental Protection Agency (EPA) standard due to wildfires.^16^

Until recently, the northeastern U.S. had been relatively unaffected by the impacts wildfires, but in June of 2023, smoke from Canadian wildfires drifted into the Northeastern U.S., resulting in multiple days of unprecedented catastrophic air quality, with PM2.5 levels reaching over 400 μg/m3 and affecting over 100 million Americans.^17^ On June 7, the Department of Environmental Pollution (DEP) monitoring sites in New York City recorded the worst air quality level in over 50 years. On June 7, 2023, New York City recorded an unprecedented daily average PM2.5 measurement of 117 μg/m^3^. The recorded value surpasses the previous record set in New York City (86 μg/m^3^), surpasses the guideline value established by the EPA by 3-fold (35 μg/m^3^), and surpasses the guideline value established by the World Health Organization (WHO) by more than 8-fold (15 μg/m^3^).^18^ The authors have sampled during this event size-fractionated PM during this Canadian wildfire event at the Rutgers campus location in New Jersey, and also confirmed that WFPM traveled almost a thousand km to reach the New York City and New Jersey densely populated areas.^19^ In the same study, the complex chemical composition of WFPM, which contains primarily organic compounds, was also confirmed.

Emissions from wildfires and biomass burning are a great concern for public health, as they contain several air pollutants, including particulate matter in the inhalable (≤10 μm, PM_10_) size range, polycyclic aromatic hydrocarbons (PAHs), which have been linked to cardiovascular diseases, poor fetal development, and carcinogenesis,^20^ other volatile organic compounds,^19,21–23^ and inorganic elements and ions, including sodium (Na), ammonium (NH^4+^), nitrate (NO^3–^), bromine (Br), chromium (Cr), iron (Fe), potassium (K), rubidium (Rb), and zinc (Zn).^24–26^ Wildfire smoke particulate matter (WFPM) consists of approximately 90% PM_2.5_, including ultrafine or nanoscale particles, PM_0.1_ (≤0.1 μm), and 10% PM_2.5–10_ (2.5–10 μm diameter).^27,28^ Significant risks to public health arise from PM_2.5_, particularly PM_0.1_, which, due to their small size, can deposit deep in the respiratory system and become systemic.^29–31^

Epidemiological studies and case reports have revealed significant impacts of wildfires on morbidity and mortality.^32–35^ In 2015, emergency room (ER) visits for patients suffering from cardiovascular disease increased significantly in California due to wildfire exposures, with increases in ER visits for myocardial infarction and ischemic heart disease of 42% and 22%, respectively.^36^ In a time series study conducted in 749 cities in 43 countries from 2000 to 2016, Chen et al. found a significant association between PM_2.5_ levels, particularly during wildfire events, and mortality from respiratory and cardiovascular diseases.^37^ Increases in PM_2.5_ from wildfires have been associated with exacerbation of asthma in children.^38,39^ More recently, three independent epidemiological studies in New York City each revealed significant increases in asthma-associated emergency visits during the June 2023 Canadian wildfire event.^40–42^

In vitro and in vivo toxicological studies of WFPM exposure have been limited to date but have begun to reveal significant potential adverse effects. For example, exposure to <1.3–2.1 μm WFPM significantly increased expression of genes associated with xenobiotic metabolism and inflammation in human bronchial epithelial cells;^43^ exposure to 0.2–10 μm WFPM collected from Helsinki in August to September of 2002 reduced viability and increased inflammatory markers in murine RAW 264.7 macrophages;^44^ and exposure to bushfire smoke extract, obtained by bubbling smoke from ignited foliage through a saline solution, caused significant cytotoxicity, reduced expression of phagocytic receptors, and diminished phagocytosis of in THP-1 and human monocyte-derived macrophages.^45^ Some of these effects could be due to the presence of PAHs and other organic functional groups, which have been linked to toxicological impacts on lung macrophages in vitro, including triggering of activation, impairing mitochondrial activity, and hindering the ability to control the growth of .^46^ The few animal studies of WFPM exposure to date have also revealed significant toxic effects in the lung. For instance, intratracheal instillation of mice with WFPM, collected in the summer of 2008 in Escalon, CA, with hydrodynamic diameters of 2.1–10.2 μm, caused a significant reduction in macrophages’ number, accompanied by increased numbers of dead cells and elevated reactive oxygen species (ROS), in bronchoalveolar lavage fluid (BALF);^47^ and mice exposed to WFPM obtained from different combustion stages (flaming vs smoldering) of various biomass fuel sources, with size distributions ranging from 32 nm to 10.57 μm, exhibited significant lung toxicity and mutagenic effects, which varied depending on fuel source at combustion stage, with the greatest effects observed from WFPM generated during the flaming phase for most fuel types.^48^ Finally, in the one relevant human exposure study to date, alveolar macrophages in BALF of individuals exposed to wood smoke for short periods of time exhibited significantly decreased viability and impaired phagocytosis and destruction of foreign particles compared to macrophages from BALF of individuals exposed to filtered air.^49^

While these initial studies have provided valuable insights into various toxicological implications of different WFPM exposures, they fall short of providing a cohesive or comprehensive assessment of WFPM toxicity. It is difficult to draw broad conclusions or gain useful mechanistic or predictive insights from this handful of studies that have employed WFMP or smoke particles from varied sources, obtained by different collection methods and having different sizes and other physicochemical characteristics, which were applied at differing doses in different experimental models and evaluated through differing experimental end points. Comparisons between such widely different studies are problematic and underscore the need for standardization of materials, methods, models, and dosimetry. Such standardization, as well as testing with environmentally relevant and fully characterized WFPM and assessment of critical toxicological end points, is needed to provide a systematic and comprehensive understanding of the potential health risks that may result from the toxicological impacts of WFPM exposure.

One potential impact of WFPM exposure that requires further investigation is the potential effect on the health and innate immune function of lung macrophages. Lung macrophages are the first line of defense against inhaled pathogens, and impairment of their ability to phagocytose and destroy pathogens could increase the risk or severity of respiratory infections. Previous studies, as noted above, have identified impaired innate immune function in THP-1- and human monocyte-derived macrophages after in vitro exposure to bushfire smoke extract and in lung macrophages recovered from human BALF after wood smoke exposure.^49^ However, no study to date has employed fully characterized real-world WFPM collected during an actual wildfire event or employed the appropriate dosimetric analyses needed to arrive at environmentally relevant doses. These limitations were addressed in the present study, in which size-fractionated and fully physicochemically characterized WFPMs collected in Piscataway, NJ, during the Canadian wildfire event of June 2023 were used to assess the impacts of WFPM exposure on human THP-1 macrophage health and function.

## 2 Materials and Methods

### 2.1 Study Design

The study design is summarized in Figure 1 and includes several novel features. First, real-world size-fractionated WFPM sampled during the June 2023 Canadian wildfire event was used to assess the effects of WFPM exposure on THP-1 macrophages. Second, to ensure that cellular doses were environmentally relevant and tethered to specific quantifiable conditions, the Multiple Path Particle Dosimetry (MPPD) model^50^ was used to calculate WFPM mass per lung surface deposition rates in the lung at relevant air WFPM exposure levels, and the distorted grid (DG) dosimetry model, previously developed by the authors,^51,54,55^ was then used to determine the corresponding administered cellular doses to match the MPPD-calculated WFPM mass per lung surface. Following exposures, cytotoxicity, viability, oxidative stress (generation of ROS), and mitochondrial membrane potential were assessed, and the binding and phagocytosis of unopsonized 1 μm polystyrene beads were quantified in exposed and control THP-1 cells.

**Figure 1:**
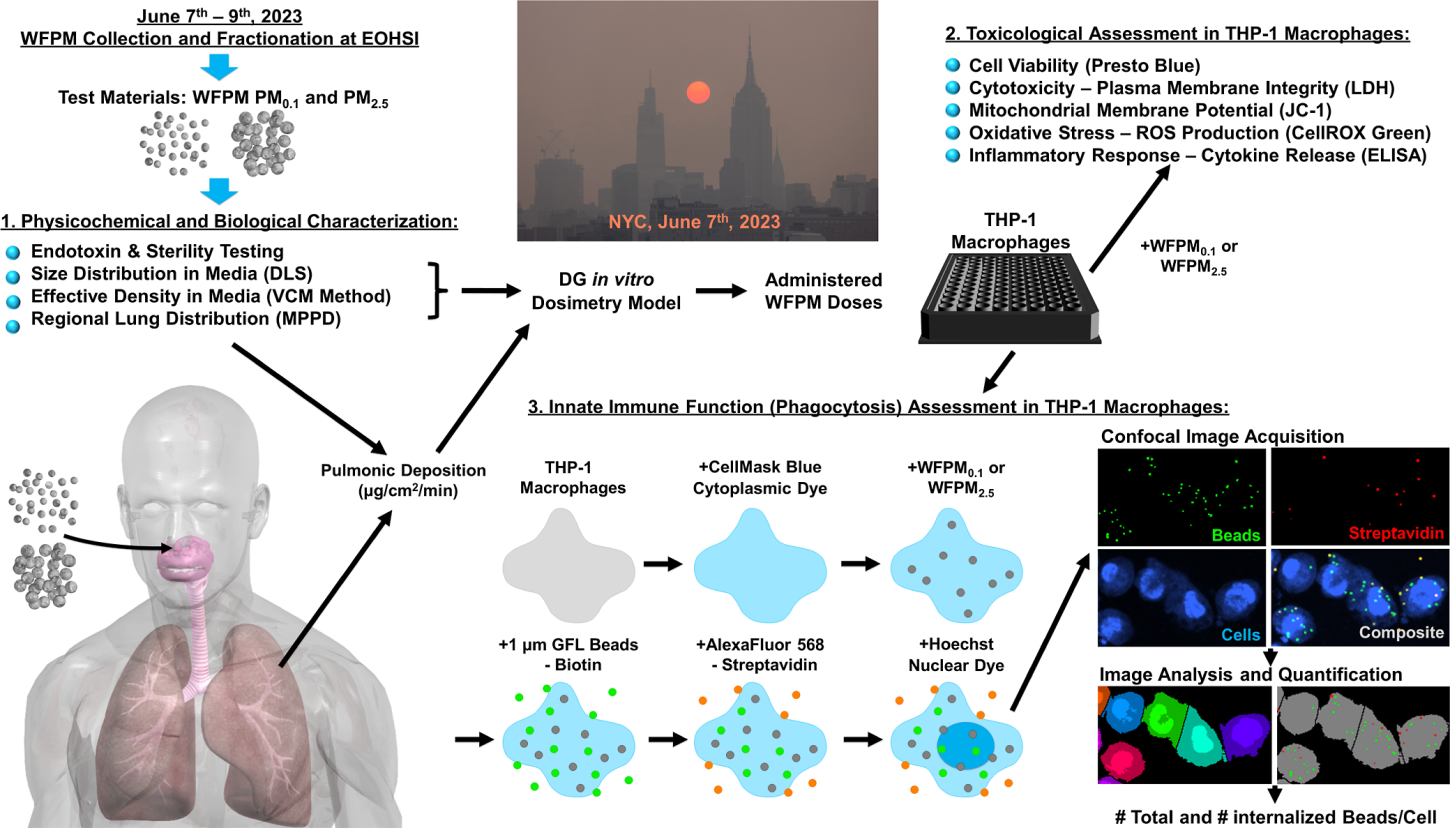
Study design overview.

### 2.2 WFPM (PM_0.1_ and PM_0.1–2.5_) Collection, Fractionation, and Extraction

Airborne size-separated WFPM samples were collected at Rutgers University’s Busch campus during the June 2023 wildfire event using the Harvard Compact Cascade Impactor (HCCI).^54^ Details on the air sampling procedure are provided in our recent publication.^19^ The HCCI consists of size-segregated impactor stages and collects particles on substrates that can be extracted and used for both physicochemical and toxicological assessment studies of PM. More specifically, the size fractions employed in this study included PM_0.1–2.5_ (0.1 to 2.5 μm), which was collected on chemically cleaned ∼0.5 × 0.5 × 4 cm filters of polyurethane foam (PUF) (Merryweather Foam, OH), as described previously in detail,^55^ and PM_0.1_ (≤0.1 μm), which was collected on precleaned 47 mm diameter polytetrafluoroethylene (PTFE) filters with a pore size of 2 μm (Pall Corporation, Port Washington, NY, USA).

Following WFPM collection, particles were extracted from the impaction substrates as previously described by the authors.^56^ Briefly, to extract WFPM_0.1_, PTFE filters were immersed, with the particle sides facing upward, in 75 mL of 75% volume/volume ethanol in a 250 mL glass beaker and subjected to bath sonication (model, Manufacturer) for 60 s. The resulting 75% ethanol particle suspension was washed five times with 75 mL of cell culture grade water (Cytiva, USA) by rotary evaporation to produce an ethanol-free aqueous suspension for toxicological studies. Efficiency for the extraction of WFPM_0.1_, calculated from gravimetric analysis of filters and dried suspensions, was ∼99%.

To extract WFPM_0.1–2.5_, PUFs were immersed in 5 mL of cell culture grade water in a 50 mL glass beaker and subjected to bath sonication for 10 min. Extraction efficiency for WFPM_0.1–2.5_, based on gravimetric analysis of PUFs and dried suspensions, was 98%.

A clean Teflon filter/PUF termed “control vehicle” (which contains no WFPM) was subjected to the above extraction and washing protocol to produce a background control solution (control vehicle) for biological experiments.^57^

### 2.3 Physicochemical Characterization of WFPM

The WFPM particles used in this study and the details on the sampling campaign and physicochemical characterization are presented in great detail in our recent publication.^19^ In summary, PM_0.1_ and PM_0.1–2.5_ were collected using the HCCI, and offline chemical characterization was completed for both particle fraction sizes as previously described in detail in a companion paper.^19^ For elemental and organic carbon analysis (EC–OC), PM_0.1_ was collected on prebaked quartz filters (Pallflex Tissuquartz filter: 47 mm diameter, Pall Corporation, Port Washington, NY). Additionally, Teflon filters (47 mm diameter and 2 μm pore size, PTFE membrane disc filters, Pall Corporation, Port Washington, NY) were used to collect PM_0.1_ for analysis via inductively coupled plasma mass spectrometry (ICP–MS). Additionally, PM_0.1_ and PM_0.1–2.5_ collected particles were analyzed for PAHs, as described by previous authors.^19,58^ ”

### 2.4 Evaluation of WFPM Endotoxin Concentration and Microbiological Sterility

Endotoxin levels in WFPM fractions and controls were measured by using the HEK-Blue LPS Detection Kit. After HEK-Blue-4 cells were incubated with samples, endotoxin standards, and spiking solutions, absorbance was measured to determine endotoxin concentrations.

Microbiological sterility was assessed by incubating samples in fluid thioglycolate medium for 14 days and inspecting for bacterial or fungal growth using PDA and PCA agar.

Additional details on methods are provided in the Supporting Information.

### 2.5 Estimation of WFPM Deposition in the Human Respiratory Tract Using the Multiple-Path Particle Dosimetry Model

The Multiple Path Particle Dosimetry (MPPD) model (V3.04) was used to calculate the mass of two size fractions of WFPM (WFPM_0.1_ and WFPM_0.1–2.5_) that would have been deposited per unit surface area (i.e., μg/cm^2^) in each major division of the respiratory system (head, tracheobronchial, and pulmonary regions) in an average human as a function of exposure time using the WFPM aerosol characterizations during the June 2023 Canadian wildfire event.^19,50,59–61^ The MPPD analysis was conducted using the methodology and parameters outlined by Lizonova et al. using the Yeh/Schum symmetric model (Schum G Yeh H-C, 1980), with a functional residual capacity of 3300 mL and a head volume of 50 mL.^53^ The nasal respiratory rate was set to 12 breaths per minute, the tidal volume to 625 mL, and the inspiratory fraction to 0.5.^62^ The aerosol input parameters used are outlined in Table S1 and were based on the PM characterization measurements that took place during the June sixth-ninth 2023 Canadian wildfire event.^19^ The effective density of WFPM was estimated using measurements reported by Lizonova et al.^53^ These data were then used to determine the suitable delivered to cell doses for conducting in vitro studies, as described below.

### 2.6 Dispersion and Colloidal Characterization for In Vitro Studies

Dispersion preparation and colloidal characterization of extracted WFPM fractions were performed as previously described by the authors.^51,52,63^

Briefly, a critical sonication energy (DSEcr) was first determined for 1 mg/mL suspensions of each WFPM fraction in cell culture grade water by subjecting suspensions to 1 min rounds of cup-horn sonication (Branson Sonifier S-450D, 400 W, with Branson 3-in. cup horn, power delivered: 1.26 W) followed by vortexing for 30 s. Hydrodynamic diameter (*z*-average, *d*_H_) and polydispersity index (PDI) were measured after each round of sonication by dynamic light scattering (DLS) using a Malvern Zetasizer Nano ZS (Malvern Panalytical Inc., Westborough, MA) until decreases in dH and PDI between rounds were negligible (<5%). Dispersions of each WFPM fraction were then prepared for use in toxicological studies by cup-horn sonication of 1 mg/mL dispersions to the corresponding DSEcr, followed by dilution of the aqueous suspension in RPMI1640 to the final administered concentrations, which were determined as described below.

### 2.7 Dosimetric Analysis and Calculation of Administered In Vitro Cellular Doses

Dosimetric analysis was performed as previously described by the authors^52,64^ to determine administered doses of each WFPM fraction that would result in delivered (deposited to cells) doses (μg/cm^2^) corresponding to various exposure time points in the pulmonic region, determined, as described above, using the MPPD model.

Here, the lower exposure duration modeled by MPPD was 4 weeks, which was typical for Canadian wildfire events during the summer of 2023. In addition, to assess effects of higher WFPM exposure concentrations and durations, doses corresponding to 10- and 100-fold higher were also evaluated for broader dose range characterization.

The effective densities of the WFPM_0.1_ and WFPM_0.1–2.5_ fractions in cell culture media (RPMI + 10% FBS) were determined using the volumetric centrifugation technique previously developed and described by the authors,^51^ and volume-weighted size distributions of each fraction were determined by DLS. For each WFPM fraction, the effective density and size distribution were then used to determine the deposition fraction (*f*_D_) and mass delivered to (deposited on) cells (μg/cm^2^) in 24 h, using the DG computational model previously developed and described by the authors.^64^ For each WFPM fraction, the administered WFPM concentration required to produce a delivered (deposited) dose (μg/cm^2^) in cell culture wells corresponding to pulmonic region deposition (μg/cm^2^) estimated by MPPD for each targeted exposure duration were then calculated as 100*Dep_MPPD_/Dep_DG_, where Dep_MPPD_ is the deposition (μg/cm^2^) calculated from the MPPD model at the target exposure duration, and Dep_DG_ is the deposition (μg/cm^2^) at 24 h at a starting (administered) dose of 100 μg/mL determined using the DG model. It is worth noting that this approach allowed us to select administered doses for the two WFPM size fractions, ensuring they matched the deposited cellular dose calculated by the MPPD model.

### 2.8 Cell Culture

THP-1 monocytes were cultured and differentiated into macrophages using the modified phorbol 12-myristate-13-acetate (PMA) (Life Technologies, Inc., Carlsbad, CA) technique suggested by Daigneault et al.^65^ Further details are provided in the Supporting Information.

### 2.9 Evaluation of WFPM In Vitro Toxicity

#### 2.9.1 Cell Viability (Mitochondrial Metabolic Activity)

The Invitrogen PrestoBlue test (Thermo Fisher, Waltham MA) was used to evaluate cell viability and mitochondrial activity, following the manufacturer’s procedure.

Briefly, following 24 h exposures of PMA differentiated macrophages to WFPM_0.1_, WFPM_0.1–2.5_, or media only (untreated 100% viable control), cells were rinsed once with PBS (200 μL/well) and then incubated at room temperature with PrestoBlue reagent (100 μL/well) for 30 min. Fluorescence intensity was quantified at excitation/emission wavelengths of 570/610 nm by using a SpectraMax M-5 microplate reader and SoftMax Pro acquisition and analysis software (Molecular Devices). Cell viability was calculated as the percentage of the signal obtained from untreated (100% viable) cells. To evaluate any possible interference caused by particles in the PrestoBlue test, both the culture medium without particles and the culture medium with particles at the maximum dosage were also analyzed by using the same assay.

#### 2.9.2 Oxidative Stress (ROS Production)

Oxidative stress was assessed by measuring cellular ROS accumulation using the CellROX green reagent (Thermo Inc., Waltham, MA) according to the manufacturer’s instructions.

Briefly, following 4 h exposure to WFPM_0.1_, WFPM_0.1–2.5_, or complete media without WFPM (negative control), or after 2 h treatment with 1.25 mM menadione (positive control), cells were rinsed with PBS and incubated with 100 μL/well of assay reaction mixture for 30 min at 37 °C. Reaction media was then removed and replaced with 200 μL of PBS, and fluorescence at ex 480/em 520 was measured using a SpectraMax M-5 microplate reader and SoftMax Pro acquisition and analysis software (Molecular Devices).

#### 2.9.3 Cytotoxicity (Membrane Damage, Lactate Dehydrogenase (LDH) Release)

Cytotoxicity (plasma membrane damage) was assessed using the Pierce LDH cytotoxicity kit (Thermo Fisher, Waltham, MA) in accordance with the manufacturer’s instructions.

Briefly, the provided LDH substrate was dissolved in 11.4 mL of ultrapure water and added to 0.6 mL of assay buffer to prepare the assay reaction mixture. Following 24 h exposure of adherent PMA differentiated macrophages to WFPM_0.1_, WFPM_0.1–2.5_, or media only (untreated control – spontaneous LDH activity), or 45 min incubation with the provided lysis buffer (positive control – maximum LDH activity), cell supernatants were collected in 1.5 mL Eppendorf tubes and centrifuged at 3000*g* for 5 min to pellet cellular debris. Fifty μL of supernatant from each tube was dispensed in triplicate wells in a fresh 96-well plate, and 50 μL of the reaction mixture was added to each well. Plates were incubated at room temperature for 30 min, and 50 mL of stop solution was added to each well to terminate the reaction. Absorbance was measured at 490 nm (*A*_490_) and 680 nm (*A*_680_) using a SpectraMax M-5 microplate reader and SoftMax Pro acquisition and analysis software (Molecular Devices). To calculate LDH activity, *A*_680_ values were subtracted from measured *A*_490_ values to correct for the instrument background. Percent cytotoxicity was calculated by subtracting spontaneous LDH release values from treatment values, dividing by total LDH activity (maximum LDH activity – spontaneous LDH activity), and multiplying by 100. To evaluate the possible background caused by particles in the Pierce LDH assay, both the culture media without particles and the culture media with particles at the maximum dosage were also subjected to the same assay.^66^

#### 2.9.4 Assessment of Inflammatory Response

The inflammatory response of PMA macrophages to WFPM was assessed by quantifying 48 cytokines and chemokines in cell supernatants after 24 h of exposure to the highest WFPM_0.1_ (36 μg/mL) or WFPM_0.1–2.5_ (8 μg/mL) doses. Cells treated with LPS (100 ng/mL) (Sigma-Aldrich, MO) for 24 h served as a positive control.

Cell supernatants were collected and transported on dry ice to Eve Technologies (Calgary, AB) for analysis of cytokines and chemokines using the Human Cytokine Array/Chemokine Array 48-Plex (HD48A) assay. The samples were prepared using Eve Technologies’ prescribed procedure. Briefly, the supernatant from each well was transferred to a 1.5 mL tube and centrifuged at 3000*g* for 10 min to eliminate cell debris. A 100 μL volume of the supernatant from each tube was then transferred to a 0.7 mL PCR tube and preserved at −80 °C until shipment. The HD48A assay measured the presence of 48 cytokines and chemokines, including granulocyte monocyte-colony stimulating factor, tumor necrosis factor-α (TNF-α), monocyte chemotactic protein 3, and interleukin-1β.

#### 2.9.5 Evaluation of Mitochondrial Membrane Potential

Effects of WFPM exposure on mitochondrial membrane potential of PMA macrophages was assessed using the JC-1 Mitochondrial Membrane Potential Detection Kit (Biotium, USA).

The green fluorescent JC-1 dye monomer accumulates and forms red fluorescent aggregates within mitochondria in a mitochondrial membrane potential-dependent manner. Mitochondrial membrane depolarization is thus indicated by a decrease in the ratio of the red to green fluorescence. The assay was performed according to the manufacturer’s protocol.

In brief, adherent macrophages were exposed to WFPM_0.1_, WFPM_0.1–2.5_, or vehicle controls for 24 h. Positive control cells were treated with 50 μM carbonyl cyanide *m*-chlorophenylhydrazone (CCCP) (Millipore Sigma, Burlington, MA) in complete RPMI at 37 °C and 5% CO_2_ for 5 min. After treatments, cells were washed with PBS and incubated with assay reagents for 15 min at 37 °C. Fluorescence was measured at 485 ex/435 em (green) and 550 ex/699 em (red) using a SpectraMax M-5 microplate reader and SoftMax Pro acquisition and analysis software (Molecular Devices). To evaluate the possible interference caused by particles in the assay, both the culture media without particles and the culture media with particles at the maximum dosage were also subjected to the same assay. Analysis was conducted by subtracting red fluorescence background (empty wells) from red fluorescence readings, and green fluorescence background (empty wells) from green fluorescence readings and calculating the ratio of red fluorescence (mitochondrial JC-1 aggregates, healthy cells) to green fluorescence (cytoplasmic JC-1 monomer) as an indicator of mitochondrial membrane potential.

### 2.10 Assessment of Macrophage Innate Immune Function

#### 2.10.1 Preparation of Unopsonized Bead Suspensions

Yellow-green fluorescent FluoSpheres Biotin-Labeled Microspheres (Thermo Fisher, Waltham, MA) with a 1.0 μm nominal diameter were utilized to prepare unopsonized bead suspensions for the phagocytosis assay (Section 2.10.2 below). Biotin-labeled beads were diluted in PBS and subjected to centrifugation for 5 min at 5000*g* to remove sodium azide. The supernatant was discarded, and pellet was resuspended in 0.5 mL of PBS. The suspension was bath sonicated for 15 min, and diluted to a final concentration of 2 × 10^8^/mL in complete RPMI1640 (without FBS) and 0.3% bovin serum albumin (BSA) (Millipore Sigma, Burlington, MA). The unopsonized particle suspension was then incubated for 15 min in a shaking incubator at 37 °C and 100 rpm.

A positive control bead suspension was prepared by adding 15 μM cytochalasin D (Cyto-D) (Millipore Sigma, Burlington, MA), an inhibitor of actin polymerization and thus of phagocytosis, to the unopsonized bead suspension.

#### 2.10.2 Phagocytosis Assay

The phagocytosis assay was conducted according to protocols previously developed and reported by the authors.^67,68^ Briefly, adherent macrophages were incubated with10 μg/mL HCS CellMaskBlue stain (Life Technologies, Carlsbad, CA) in complete RPMI1640 (Life Technologies) for 40 min at 37 °C and 5% CO_2_. The dye solution was then removed and cells were incubated with WFPM_0.1_, WFPM_0.1–2.5_, or vehicle controls suspensions for 24 h at 37 °C and 5% CO_2_. Positive control wells were incubated with 15 μM cytochalasin D (Millipore Sigma) in complete RPMI1640 for 30 min. Cells were then washed with PBS and incubated with unopsonized bead suspension alone or with 15 μM cytochalasin D (positive control) for 40 min for binding and internalization of beads. Macrophages were then washed with cold PBS, incubated with 5 μg/mL AlexaFluor568-streptavidin (Life Technologies, Inc., Carlsbad, CA) in PBS + 1% BSA at 4 °C for 30 min, rinsed twice with cold PBS, and fixed with 4% formaldehyde at room temperature for 10 min. Cells were then washed with PBS and incubated with 2 μg/mL Hoechst 33342 nuclear dye (Life Technologies, Inc., Carlsbad, CA) in PBS (100 μL/well) at room temperature for 60 min. Cells were then washed once with PBS and stored with 200 μL/well of fresh PBS at 4 °C prior to confocal imaging.

#### 2.10.3 Confocal Fluorescence Microscopy and Image Analysis

Confocal fluorescence image z-stacks were acquired by using an SP8 LIGHTNING confocal microscope (Leica Microsystems, USA). Three image color channels were acquired for each well: CellMask Blue and Hoechst nuclear stain (blue); all beads (green); and external beads labeled with AlexaFluor568-streptavidin (red). Processing and analysis of confocal images was performed using custom MATLAB software (The MathWorks, Inc., Natick, MA, USA) developed and previously described in detail by the authors.^67,68^

Briefly, the MATLAB software uses the blue fluorescence channel images (CellMask blue cytoplasmic stain and Hoechst nuclear stain) to segment and identify individual cells, the green fluorescence channel images (green fluorescent beads) to segment and identify all individual fluorescent beads (internal or external to cells), and the red fluorescence channel images (AlexaFluor568-streptavidin) to segment and identify individual external beads (not within cells and thus accessible to binding by streptavidin). The software then determines bead-cell associations by identifying all bead (green channel) and external bead (red channel) objects sharing pixels with cell objects (blue channel). Bead objects associated with cells are classified as “external” if they overlap with bead objects in the red channel and are otherwise labeled as “internal”. The software then calculates the mean total number of beads bound or internalized per cell (internal + external beads) and the mean fraction of beads internalized by cells (internal/(internal + external)). This analysis was performed for each treatment and control condition.

### 2.11 Statistical Analysis

All toxicity studies were conducted in triplicate plates (biological repeats), with three technical replicates (wells) in each plate for each treatment. Statistical analysis was completed, and graphs were generated using Prism 10 software for a MacBook (GraphPad Software, Inc., San Diego, CA). Statistical significance of differences between untreated, vehicle control, and positive controls in toxicological and innate immune function assays was analyzed using paired *t* tests. Statistical significance of differences between vehicle control and WFPM exposures at each dose for all assays was assessed by one-way ANOVA with Tukey’s multiple comparison tests.

## 3 Results and Discussion

### 3.1 Physicochemical Characterization of WFPM

Physicochemical characterization of both WFPM fractions has been reported in great detail in a companion paper by Cedeño Laurent et al.^19^ In summary, elemental and organic carbon (EC/OC) analysis of WFPM_0.1_ revealed a preponderance of organic carbon (OC of 139.7 μg/m^3^ compared to EC at 6.1 μg/m^3^), with an organic to total carbon (OC/TC) ratio of 0.96. This pattern is in alignment with signatures typically associated with biomass burning.^23^

PAH analysis for both WFPM fractions showed a cumulative PAH mass concentration of 98.1 ng/m^3^, representing varying proportions in the two WFPM size fractions: 14.1% WFPM_0.1_ and 41.3% of WFPM_0.1–2.5_. The majority of PAHs was made up of high-molecular-weight PAHs (e.g., Retene), which is associated with higher cellular toxicity and genotoxicity.^69^ In addition, Retene was found at a higher concentration in WFPM_0.1–2.5_ compared to WFPM_0.1_ (Figure S1).

Elemental analysis of WFPM_0.1_ identified 12 inorganic elements, which included both crustal elements such as Fe, Mn, and Al, which originate in the Earth’s crust, and metals such as Ba, Ti, Cr, Zn, Pb, Sn, Ni, Sb, and Cu, which are associated with anthropogenic sources (Figure S2).

### 3.2 Microbiological and Endotoxin Analysis

Following a 14 day period of incubation, the presence of microorganisms was seen on agar plates for the wildfire fractions WFPM_0.1_ and WFPM_0.1–2.5_. No microorganisms were detected in vehicle controls. These results are expected, as WFPM fractions were collected from ambient air, which contains a variety of microorganisms, including bacteria and fungi.

No detectable levels of endotoxin were found in the WFPM_0.1_ samples or vehicle controls, all of which were below the limit of detection of the test. Analysis of WFPM_0.1–2.5_ revealed an endotoxin concentration of 0.039 EU/mL, which is well below the allowable limit of 0.1 EU/mL for cell culture studies.

### 3.3 Colloidal Characterization of WFPM Suspensions in Culture Medium

The DSEcr for each WFPM size fraction was determined as described in Section 2.4. Detailed colloidal characterization of WFPM_0.1_ and WFPM_0.1–2.5_ dispersions in water and in RPMI1640 (at *t* = 0 and 24 h) are presented in Table S2. In summary, the hydrodynamic diameter (*d*_H_) and PDI of WFPM_0.1_ in water remained stable regardless of sonication energy applied (*d*_H_ = 242.9 ± 71.5, PDI = 0.081 ± 0.017). The DSEcr for WFPM_0.1_ was therefore 0 J/mL, meaning that no sonication of WFPM_0.1_ aqueous suspensions was required before dilution in media to prepare exposure suspensions. In contrast, WFPM_0.1–2.5_ had a DSEcr of 2179.6 J/mL (yielding a dispersion with *d*_H_ = 532.4 ± 43.84 nm, and PDI = 0.497 ± 0.092).

Suspensions of both WFPM fractions in water after sonication to DSEcr remained relatively stable for 24 h. Both WFPM fractions exhibited negative zeta potential values (WFPM_0.1_: −34.63 ± 1.43 mV, WFPM_0.1–2.5_: −32.3 ± 1.10 mV), indicating effective electrostatic stabilization of particles and ensuring a favorable colloidal dispersion. Suspensions of both WFPM fractions in complete RPMI1640 media had a positive zeta potential of ∼9 mV. The increase in zeta potential upon dispersion in culture media can be attributed to the adsorption of negatively charged serum proteins on the surface of the particles and the formation of a protein corona.

The mean effective densities (ρ_EV_) of WFPM_0.1_ and WFPM_0.1–2.5_ in complete RPMI were calculated as described in the methods and were found to be 1.565 ± 0.07 and 1.65 ± 0.0 g/cm^3^, respectively. The bulk density of WFPM was assumed to be 1.7 g/cm^3^, as previously determined by the authors.^70^

### 3.4 In Vitro and In Vivo Dosimetric Calculations

The deposition rates (μg/cm^2^/min), total depositions (μg/cm^2^) for each of wildfire exposure duration (from MPPD), corresponding *f*_D_, in vitro delivered cell culture dose (from DG model) and in vitro administered concentrations required to match the 40 week deposition for each WFPM fraction are presented in Table 1. The pulmonic MPPD model deposition rate (μg/cm^2^/min) of the smaller WFPM_0.1_ fraction was 4.567 × 10^–7^ μg/cm^2^/min, more than twice that of the WFPM_0.1–2.5_ fraction (1.940 × 10^–7^ μg/cm^2^/min), while the 24 h in vitro DG model deposition fraction of the smaller WFPM_0.1_ (∼11%) was roughly half that of the WFPM_0.1–2.5_ fraction (∼21%). The greater in vitro deposition of WFPM_0.1–2.5_ is attributed to its larger particle size and thus faster settling rate in comparison to that of WFPM_0.1_. Because of its roughly 2-fold slower settling in culture media and nearly 2-fold greater deposition in the lung, the matching delivered in vitro dose (DG model) required to match the 40 week exposure lung deposition (MPPD model) for WFPM0.1 (1.98 × 10^–3^ μg/cm^2^) was about 2-fold greater than that for WFPM_0.1–2.5_ (8.38 × 10^–4^ μg/cm^2^). Each WFPM size fraction was matched to its respective administered concentration to match the same delivered to cell dose, reflecting the different deposition dynamics.

**Table 1:** Dosimetry and In Vitro Dose Calculations^a^

	MPPD deposition rate/min (μg/cm^2^/min)	MPPD 40 weeks deposition (delivered target) (μg/cm^2^)	DG 24 h deposition fraction	delivered dose to match MPPD (μg/cm^2^)	administered dose (concentration) to match MPPD (μg/mL)
WFPM_0.1_	4.567 × 10^–7^	1.976 × 10^–3^	0.113	1.98 × 10^–3^	3.6
WFPM_0.1–2.5_	1.940 × 10^–7^	0.838 × 10^–3^	0.206	8.38 × 10^–4^	0.8

a Deposition rates and 40 week depositions from MPPD, deposition fractions (*f*_D_) in in RPMI + 10% FBS determined from the DG model, and delivered in vitro doses matching 40 week deposition for WFPM_0.1_ and WFPM_0.1–2.5_.

Three doses of WFPM_0.1_ and WFPM_0.1–2.5_ were administered, with the lowest dose representing a 4 week exposure period as certain wildfires and locals (i.e., West coast USA wildfire events) can last for several weeks and months.^71–74^ To assess the full dose–response range, 10-fold and 100-fold higher doses were also used. Although the 10- and 100-fold doses of wildfire smoke exposure may be unlikely in most places, it may be relevant for people who live in the proximity of the fires, whereas higher PM concentrations occur or persist for prolonged periods and who are therefore chronically exposed to wildfire smoke. In addition, high doses may closely simulate exposures of firefighters and other first responders working directly in the wildfire area, who would be exposed to much greater wildfire smoke concentrations for shorter times.”

### 3.5 Toxicological Evaluation of WFPM

Results of in vitro toxicity assessment of WFPM_0.1_ and WFPM_0.1–2.5_ in PMA-differentiated THP-1 macrophages is summarized in Figure 2 and described in detail below. It is worth noting that the administered doses (concentration) for the two size fractions were calculated as described in the Materials and Methods section to match the same delivered to cell doses for the two size fractions.

**Figure 2:**
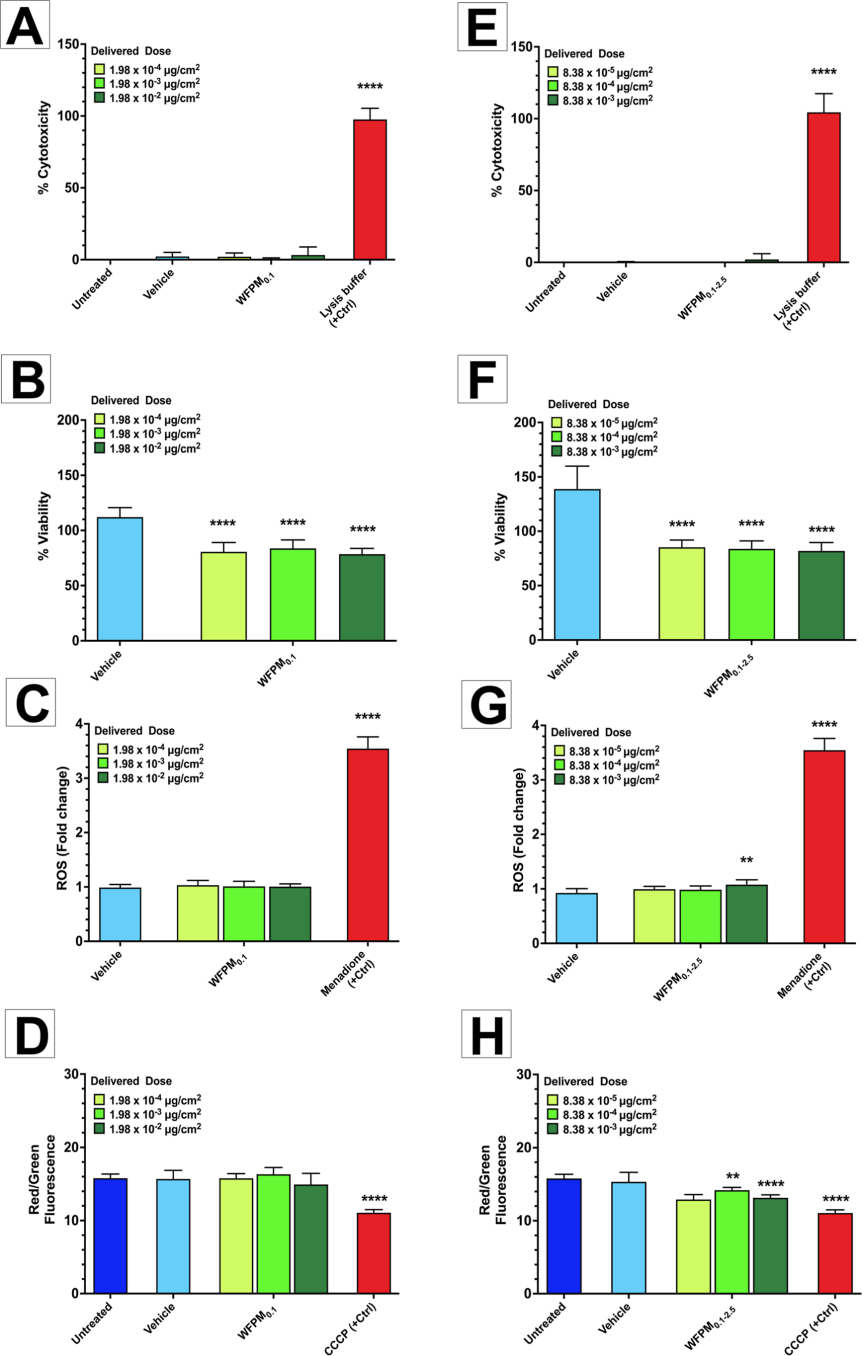
Evaluation of the acute toxicological impacts of WFPM_0.1_ and WFPM_0.1–2.5_ on THP-1 differentiated macrophages as a function of administered WFPM doses. (Note: the administered doses for the two WFPM size fractions were selected to match the same delivered to cell dose calculated by the MPPD model). (A) Cytotoxicity assessed by quantification of extracellular release of LDH (plasma membrane damage) after exposure to WFPM_0.1_ or vehicle control for 24 h. (B) Cell viability (mitochondrial enzyme activity) assessed using the PrestoBlue assay following exposure to WFPM_0.1_ or vehicle control for 24 h. (C) Intracellular production of ROS quantified using the CellROX Green assay after 4 h exposures to WFPM_0.1_ or vehicle control. (D) Mitochondrial membrane potential assessed using the JC-1 Mitochondrial Membrane Potential Detection Kit after exposure to WFPM_0.1_, vehicle control, or CCCP (positive control) for 24 h. (E) Cytotoxicity assessed by quantification of extracellular release of LDH (plasma membrane damage) after exposure to WFPM_0.1–2.5_ or vehicle control for 24 h. (F) Cell viability (mitochondrial enzyme activity) assessed using the PrestoBlue assay following exposure to WFPM_0.1–2.5_ or vehicle control for 24 h. (G) Intracellular production of ROS quantified using the CellROX Green assay after 4 h exposures to WFPM_0.1–2.5_ or vehicle control. (H) Mitochondrial membrane potential assessed using the JC-1 Mitochondrial Membrane Potential Detection Kit after exposure to WFPM_0.1–2.5_, vehicle control, or CCCP (positive control) for 24 h. (*N* = 3. **p < 0.01, ****p < 0.0001).

### 3.6 Cytotoxicity (LDH Release)

Cytotoxic effects, as measured by LDH release (plasma membrane damage), of WFPM exposure on PMA macrophages are summarized in Figure 2A,E. Neither WFPM_0.1_ or WFPM_0.1–2.5_ caused significant cytotoxicity at any dose after 24 h exposure. Treatment with lysis buffer (positive control, 100% toxicity) strongly and significantly increased LDH release (*p* < 0.001), as expected.

### 3.7 Cell Viability (Mitochondrial Enzyme Activity)

Effects of WPFM exposure on PMA macrophage cell viability (mitochondrial enzyme activity), as measured by the PrestoBlue assay, are summarized in Figure 2B,F. WFPM_0.1_ exposure reduced viability by ∼20% (*p* < 0.001) compared to vehicle controls at all experimental doses. Similarly, WFPM_0.1–2.5_ decreased viability by ∼38% (*p* < 0.001) at all experimental doses compared to vehicle controls. These results might be attributable to the presence of PAHs and other organic and inorganic compounds found in both wildfire fractions reported by Cedeño Laurent et al.^19^ In particular, high-molecular-weight PAHs, which are associated with higher cell toxicity compared to low-molecular-weight PAHs, were found in much greater amounts than low-molecular-weight PAHs in both WFPM fractions (Figure S1).^19^ Inorganic elements detected in WFPM_0.1_ samples (e.g., Fe, Ba, Pb), represented in Figure S2, could also have contributed to the observed reduction in cellular viability.^19^ PM containing PAHs and metals from ambient and occupational exposures have been found to have genotoxic properties, which could result in direct damage to DNA, which in turn could trigger cell cycle arrest, apoptosis, or cellular senescence, ultimately leading to decreased viability.^75,76^ Overall, these viability results are in agreement with those of previous studies of macrophages exposed to biomass PM containing PAHs. Exposure of PMA differentiated macrophages to collected woodsmoke (containing PAHs) was previously found to cause a significant decrease in cellular viability.^46^ Moreover, Franzi et al. reported a significant decrease in cellular viability in murine macrophages after 24 h exposure to WFPM collected in June 2008 from a rural area in the San Joaquin Valley.^47^

### 3.8 Oxidative Stress (ROS Production)

Results of assessment of oxidative stress (ROS production) in PMA-differentiated THP-1 macrophages after exposure to three delivered doses of the two WFPM size fractions for 4 h are shown in Figure 2C,G. Only the highest dose of WFPM_0.1–2.5_ resulted in a significant increase (∼15%, *p* < 0.01) in ROS compared to untreated cells. Neither WFPM_0.1–2.5_ at the two lower doses, WFPM0.1 at any of the three doses, nor vehicle controls caused a significant increase in ROS production. The positive control (menadione) significantly increased ROS (*p* < 0.0001) compared to untreated and vehicle controls, as expected. The increase in ROS at the high dose of WFPM_0.1–2.5_ but not WFPM_0.1_ might be attributable to the higher concentrations of PAHs present in the WFPM_0.1–2.5_ fraction compared to the WPFM_0.1_ fraction (40.5 ng/m^3^) in WFPM_0.1–2.5_ vs 13.8 ng/m^3^ in WFPM_0.1_, as reported by Cedeño Laurent et al.^19^ These results are in agreement with previous studies, which demonstrated that the presence of PAHs in ambient particles can cause an increase in oxidative stress.^77^ However, due to the presence of other toxic compounds, such as redox-active metals, more detailed studies are needed to specifically attribute PAHs or other toxic compounds present in WFPM as the causative agents underlying the observed toxicity. For example, the redox-active metals found in WFPM may have also altered the redox status of cells by disrupting the balance between ROS production and antioxidant defenses, leading to an increase in oxidative stress, cellular damage, and altered immune responses.^79^

### 3.9 Mitochondrial Membrane Potential

Effects of WFPM exposure on PMA macrophage mitochondrial membrane potential, represented as the ratio of red to green fluorescence in the JC-1 assay (described in methods Section 2.9.5) are summarized in Figure 2D,H. Both WFPM fractions caused a significant decrease (∼15%, *p* < 0.001) in mitochondrial potential (red/green fluorescence ratio) relative to vehicle controls at the highest dose but had no effect at either of the lower doses. The positive control treatment (CCCP) strongly decreased mitochondrial potential (∼30%, *p* < 0.001), as expected.

It is also worth noting that neither WFPM fraction caused any interference with the fluorescence intensity in the endotoxin, cytotoxicity, PrestoBlue, ROS, and membrane potential assays (data not shown).

### 3.10 Inflammatory Response (Cytokines and Chemokine Release)

The inflammatory responses in PMA differentiated THP-1 macrophages after 24 h exposures to WFPM_0.1_ or WFPM_0.1–2.5_ were assessed by quantitative analysis of cytokines and chemokines in cell supernatants as described in methods Section 2.9.4. No significant differences were observed between vehicle controls and WFPM_0.1_ or WFPM_0.1–2.5_ treatments for any of the 48 cytokines and chemokines analyzed. Treatment with LPS (positive control) produced a strong and significant increase (*p* < 0.0001) in all six selected cytokines/chemokines, as expected (data not shown).

The observed outcomes might be ascribed to the relatively low administered doses of WFPM_0.1_ (36.0 μg/mL) and WFPM_0.1–2.5_ (8 μg/mL) utilized in this study compared to previous studies. These concentrations are considerably lower than those employed in previous studies in which significant changes in cytokines were observed in macrophages after exposure to WFPM. Significant increases in release of pro-inflammatory cytokines such as IL-6 and TNF-α have been observed in primary alveolar macrophages exposed to ambient air pollution particles at a 50–100 μg/mL for 20 h.^80^ Likewise, significant increases in concentrations of IL-6, TNF-α, and MIP-2 were observed in bronchoalveolar lavage fluid from mice exposed to 50–100 μg of WFPM by intratracheal instillation.^48^ Although these studies showed significant inflammatory responses, it is important to note that the doses used were considerably greater than those used in our studies.

### 3.11 Effects of WFPM on Innate Immune Function (Phagocytosis)

Using the differential staining approach and confocal imaging described in methods Section 2.10, we examined the effect of WFPM exposure on phagocytosis of unopsonized fluorescent polystyrene beads. Representative composite confocal images of bead uptake by PMA-differentiated THP-1 macrophages are shown in Figure 3, where beads external to cells appear as orange-yellow (due to colocalization of green signal from the beads and red signal from bound AlexaFlour568-streptavidin), and internalized beads appear as green only. The highest level of bead uptake (greatest number of green only beads per cell) was seen in untreated cells (Figure 3A). Uptake appeared to be greatly reduced after treatment with the positive control cytochalasin D (Figure 3B), as expected. Treatment with WFPM_0.1_ or WFPM_0.1–2.5_ also appeared to reduce internalization of beads relative to that of the untreated sample (Figure 3C,D).

**Figure 3:**
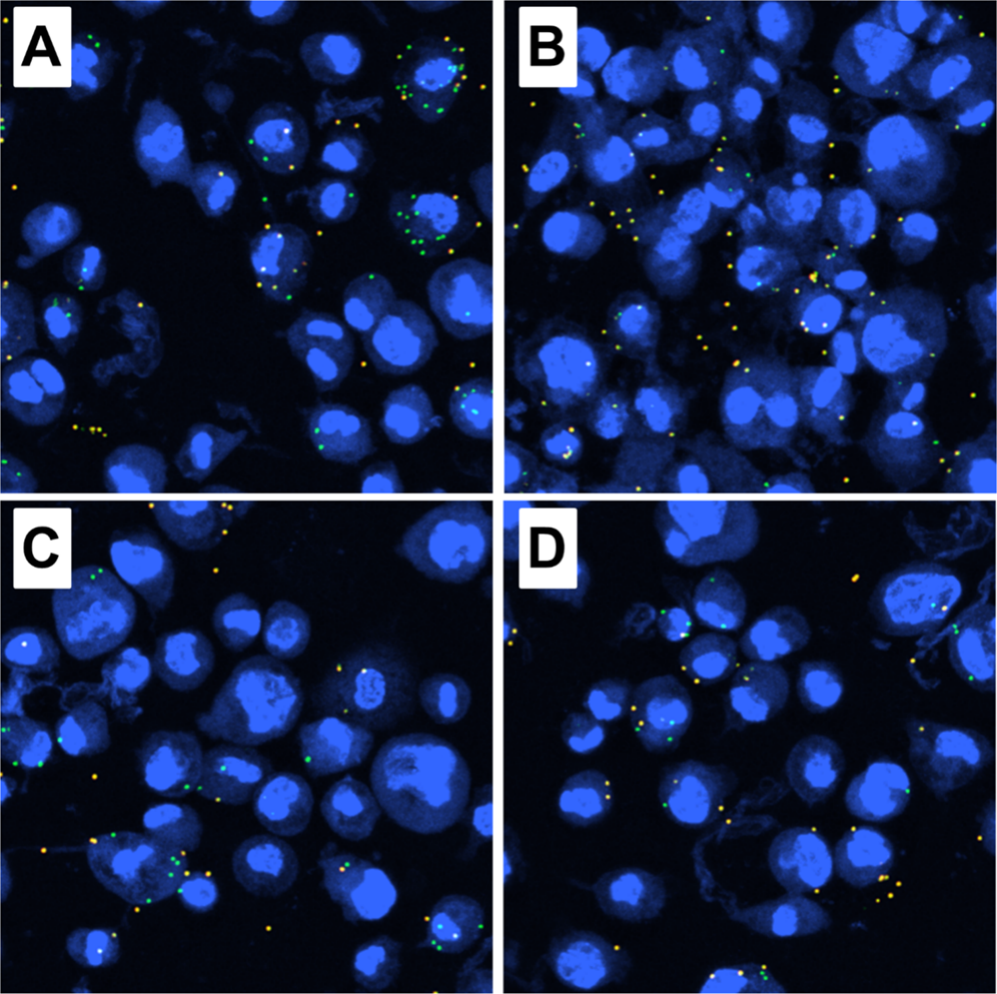
Assessment of fluorescently labeled polystyrene bead phagocytosis following WFPM exposure. Adherent PMA-differentiated THP-1 macrophages were incubated for 24 h with WFPM_0.1_ or WFPM_0.1–2.5_ prior to incubation with unopsonized 1 μm biotinylated green fluorescent polystyrene beads. Representative composite confocal fluorescence images from polystyrene bead phagocytosis experiments are shown. All beads are seen in the green channel, external beads, labeled with streptavidin-AlexaFluor 594, are seen in the red channel, and CellTracker Blue cytoplasmic stain and Hoechst nuclear dye are seen in the blue channel (cells). (A) Untreated (media only). (B) Cyto-D (positive control). (C) WFPM_0.1_ (delivered dose: 1.98 × 10^–3^ μg/cm^2^). (D) WFPM_0.1–2.5_ (delivered dose: 8.38 × 10^–4^ μg/cm^2^).

Results of quantitative analysis of confocal images (using our custom MATLAB software described in methods Section 2.10.3) are shown in Figure 4. Both doses of both WFPM_0.1_ and WFPM_0.1–2.5_ resulted in a significant decrease (∼31%, *p* < 0.0001) in the average total number of beads bound and/or internalized per cell compared to vehicle and untreated controls (Figure 4A,C). Likewise, both doses of WFPM_0.1_ and WFPM_0.1–2.5_ significantly decreased (∼34%, *p* < 0.0001) the average number of internalized beads per cell (Figure 4B,D). Treatment with cytochalasin D (positive control) reduced the number of beads internalized per cell by ∼50% (*p* < 0.001) as expected.

**Figure 4:**
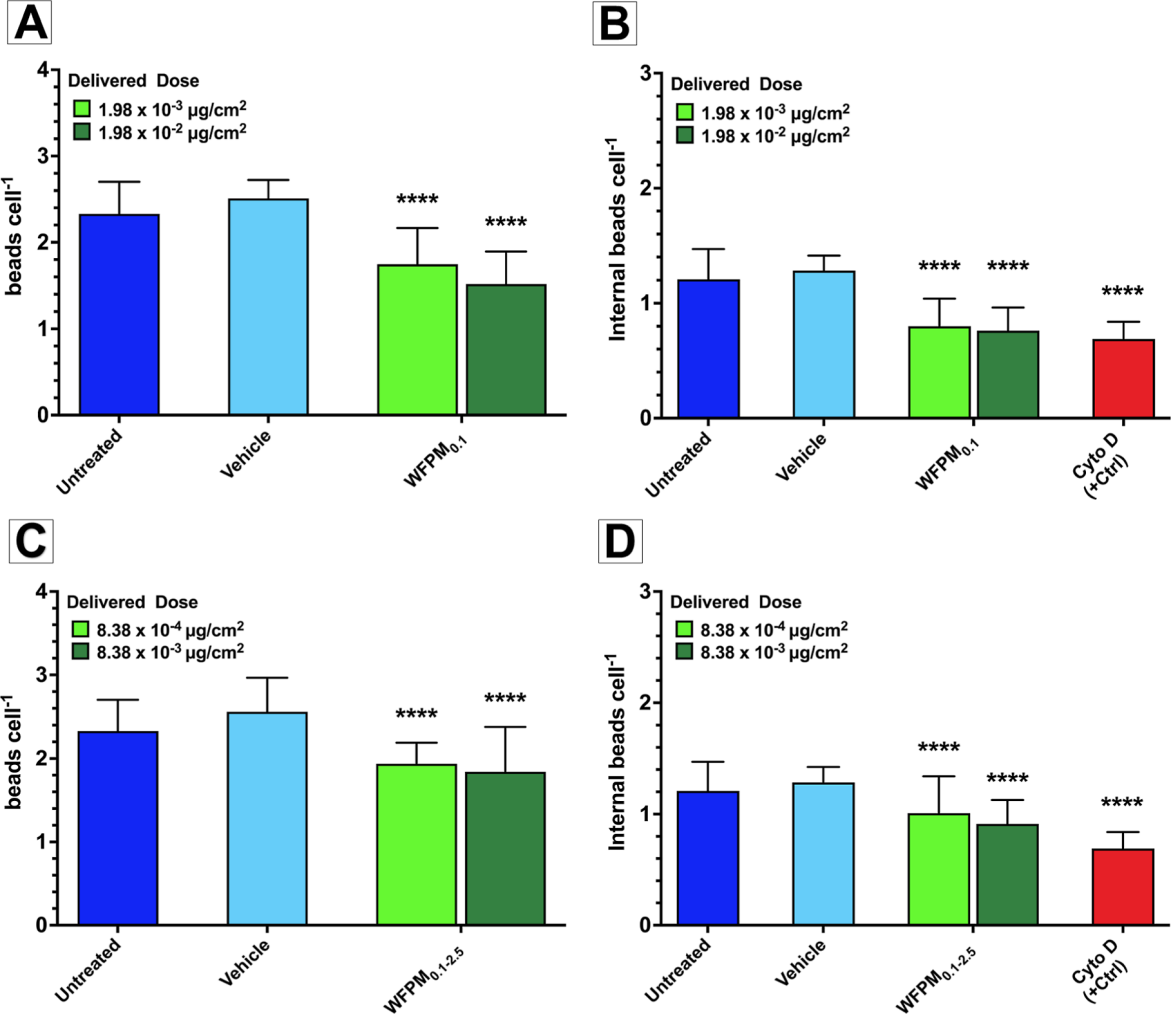
Quantification of fluorescent polystyrene bead phagocytosis following WFPM exposure. Adherent PMA-differentiated THP-1 macrophages were incubated for 24 h with WFPM_0.1_ or vehicle control, or for 30 min with cytochalasin D (Cyto D, positive control), or were left untreated prior to incubation with unopsonized 1 μm biotinylated green fluorescent polystyrene beads. (Note: the administered doses for the two WFPM size fractions were selected to match the delivered cell doses calculated by the MPPD model). (A) Total number of beads per cell (internalized + external bound) following WFPM_0.1_ exposure. (B) Number of internalized beads per cell following WFPM_0.1_ exposure. (C) Total number of beads per cell (internal + external bound) following WFPM_0.1–2.5_ exposure. (D) Number of internalized beads per cell following WFPM_0.1–2.5_ exposure. (*N* = 3. ****p < 0.0001).

An advantage of the confocal imaging and analysis methodology employed in this study compared to commercial plate reader-based phagocytosis assays is that it provides the numbers, on a per cell basis, of both particles internalized and particles bound but not internalized (as well as total-bound + internal), which allows insight into the mechanisms underlying an observed impact on phagocytosis. Specifically, the ratio of internalized beads to total (bound + internal) beads reveals the relative contributions of defects in binding (receptor expression, integrity, or availability) and defects in the cellular signaling pathways and cytoskeletal processes involved in phagocytosis.^67,68,81^ Since each internalized particle must first have been bound, a primary defect in binding alone would be characterized by identical decreases in total and internalized beads, whereas a defect in internalization alone would present as a decrease in internalized beads alone, with no change in total beads per cell. In the case of a binding defect alone, the ratio of internalized to total beads per cell would therefore be unaffected (identical to controls), whereas in the case of an internalization defect alone, that ratio would be significantly decreased. In our results, WFPM exposures reduced both total and internalized beads to almost identical extents, though the effect on number internalized beads (34%) was slightly greater than the effect on total beads (31%). The observations that both the number of total beads and number of internalized beads were decreased by nearly the same percentage and that the ratio of internalized to total beads was not significantly changed compared to controls (data not shown) suggest that the primary defect caused by WFPM exposure was a binding defect. Since scavenger receptors are the primary phagocytic receptors involved in uptake of unopsonized environmental particles,^82^ this could be the result of either impaired transcription, translation, modification, or trafficking of scavenger receptors; or of binding and blocking of scavenger receptors by WFPM particles. It has been previously reported that exposure of macrophages to WFPM can significantly impair innate immune function by altering expression of phagocytic recognition receptors.^45^ Specifically, WFPM exposure resulted in a significant reduction in the percentage of cells expressing scavenger receptors.^83^ It is therefore possible that the observed reduction in bead phagocytosis observed after WFPM exposure in this study was in part due to the reduced expression of scavenger receptors, which in turn could result from the observed diminished cell viability (metabolic enzyme activity). While these results identified binding as the primary defect underlying the impairment of bead phagocytosis, identifying the specific contributions of receptor blockade and transcription, translation, post-translational modification, or trafficking of receptors will require further study, which is underway in our lab.

Although the primary focus of this study is to investigate the toxicity of collected WFPM exposed to THP1 macrophages, it is inevitable that the WFPM fractions may also contain PM from sources other than wildfires. These additional sources may include PM emissions from traffic and industrial activities, which can include heavy metals and other organic species, all of which are known to contribute to cellular toxicity and macrophage dysfunction.^70^ While it is difficult to source abortion and quantify the exact amount of particles solely from the wildfires, it is worth noting that as shown from our previous publication on the Canadian wildfire event,^19^ the mass fraction of PM2.5 increased by a factor of ∼110 from 3 μg/m^3^ before the Canadian wildfire event to 330 μg/m^3^ afterward.

It is also worth noting that the physicochemical properties of WFPM varies and is a function of wood species burned and other wildfire conditions.^23,84^ This study focuses solely on the Canadian wildfire event and the WFPM particles sampled and physicochemically characterized in great detail in our companion study.^19^

While this study evaluates the toxicity of WFPM by applying them directly to macrophages in cell culture, in real-world inhalation exposures, inhaled WFPM would first interact with the respiratory epithelium and fluids in the oropharynx and nasopharynx, trachea, and 23 branches of airways before reaching the alveoli and alveolar macrophages. In the course of these interactions, the physicochemical properties of the WFPM could be altered, and some of the chemical components of WFPM could be metabolized, potentially altering their toxicity. For instance, PAHs, which are a predominant component WFPM, have been found to activate phase I and II cytochrome P450 (CYP) enzymes.^85^ These enzymes, regulated by both AhR-dependent and independent pathways, facilitate the metabolism and detoxification of PAHs, but can also generate reactive intermediates that contribute to oxidative stress and inflammation, influencing the toxicity of WFPM.^78^ These in vivo processes play a critical role in determining the overall health implications of WFPM and need to be considered alongside in vitro results for a more complete assessment of their toxicity by using in vivo animal studies.

In conclusion, our study highlights the significant impact of WFPM on lung macrophage function, demonstrating that exposure to WFPM can impair phagocytosis, increase oxidative stress, and decrease cellular viability, potentially increasing susceptibility to inhaled pathogens. These findings underscore the need for further investigations to evaluate the effects of WFPM exposure on the innate immune function of lung macrophages, including phagocytosis, under both opsonized and unopsonized conditions (which proceed via different receptors and signaling pathways) of environmental particles, bacteria, fungi, and yeast. While unopsonized phagocytosis, mediated by scavenger receptors, plays a key role in the lung, opsonized phagocytosis—mediated by Fc and CR3 receptors, which recognize particles or microorganisms tagged with antibodies or complement—also plays a crucial role.^86^ For example, opsonized phagocytosis is essential for effective uptake and clearance of encapsulated bacteria such as (Pneumococcus).^87^

In addition, future studies should also include in vivo investigations and mechanistic studies of WFPM exposure to provide a more complete understanding of the potential role of WFPM exposure in the development of various negative pulmonary outcomes. In addition, the integration of pharmacokinetics with toxicity studies is crucial for understanding the uptake, distribution, metabolism, and elimination of WFPM and their chemical constituents in the body.

It is also worth noting that the use of “real world”, environmentally relevant doses in studies of the health effects of WFPM is essential for accurately assessing risks, enhancing translational relevance, and promoting consistency in research findings. By employing doses that reflect real environmental conditions, researchers can provide valuable insights into the real potential health impacts of wildfire events and inform strategies to protect public health.

Finally, findings from our study confirm that WFPM can travel long distances and persist in the environment for extended periods and can affect the health of people in densely populated metropolitan areas such as the New York City area. Comprehensive toxicological studies like these are essential for informing public health assessors and help in developing enhanced public health guidelines and recommendations for wildfire incidents.

## Supplementary Material



## Notes

Methods; results of mass concentration of PAHs in WFPM fractions; results of elemental analysis of WFPM_0.1_; MPPD input parameters; and results of colloidal characterization of WFPM in water and cell culture medium (PDF)
