# Supplementary material for: Impact of Canadian Wildfire-Emitted Particulate Matter on THP‑1 Lung Macrophage Health and Function
Source: Environ Sci Technol. 2025 Feb 18;59(8):3869–83. doi: 10.1021/acs.est.4c10304 (PMC12036631; doi:10.1021/acs.est.4c10304)
Supplement: Supplementary file 1 [file es4c10304_si_001.pdf]

Supporting Information

Number of Pages: 22
Number of Figures: 2
Number of Tables: 2

Impact of Canadian wildfire-emitted particulate matter on THP-1 lung macrophage health and function

Lila Bazina [a,b], *Glen Deloid* [a], *Luke Fritzky* [c], *Denisa Lizonova* [a], *Nachiket Vaze* [a],

and Philip Demokritou [a,b*]

a. Nanoscience and Advanced Materials Center, Environmental and Occupational Health Sciences Institute (EOHSI), Rutgers University, Piscataway, NJ 08854, USA

b. Department of Environmental Occupational Health and Justice, School of Public Health, Rutgers University, Piscataway, NJ 08854, USA

c. New Jersey Medical School, Cancer Institute of New Jersey, Rutgers University , Newark, NJ 07103,USA

*Email: philip.demokritou@rutgers.edu

Supplementary Methods

Evaluation of WFPM endotoxin concentration and microbiological sterility

Endotoxin levels in the final $WFPM_{0.1}$ and $WFPM_{0.1\text{-}2.5}$ fraction suspensions, and in filter and PUF extraction control solutions, were assessed using the HEK-Blue™ LPS Detection Kit 2 (Invivogen, San Diego, CA, USA) according to the manufacturer's instructions. HEK-Blue™-4 cells were grown in Dulbecco's modified Eagle's High Glucose medium (DMEM, Life Technologies, Inc., Carlsbad, CA) without phenol red, supplemented with 10% heat-inactivated ultra-low endotoxin fetal bovine serum (FBSLE, Corning, Inc.), 2 mM L-alanyl-L-glutamine (Corning, Inc.), 100 U/mL penicillin and 100 μg/mL streptomycin (Life Technologies, Inc., Carlsbad, CA),100 μg/mL Normocin, and the provided selection antibiotics. Briefly, 20 μL of 100 μg/mL suspensions of each sample was dispensed into designated wells of a tissue culture treated 96 well plate. Endotoxin free water was added to some wells for background and calibration measurements. Endotoxin standards from Escherichia coli serotype 055:B5 (0.01–1 EU/mL), and spiking solution (0.1 EU/mL) were also prepared and dispensed into designated wells. A suspensions of HEK-Blue™-4 cell suspension (160 μL containing 48,000 cells) was then added to all wells and the plate was

incubated for 20 h at 37˚C in 5% CO_2. Supernatants (40 μL) from the incubated plate were then transferred to a new detection plate, and 160 μL of Quanti-Blue (QB) reagent mixture was added to all wells. The plate was then incubated at 37˚C until color development was complete (2-4 h). Absorbance was measured at 620 nm and endotoxin concentration was determined from the standard curve created from absorbances measured in the endotoxin standard wells. WFPM samples spiked with an additional 0.1 EU/mL of endotoxin were used to assess potential interference.

The microbiological sterility of all WFPM fractions and control vehicles utilized were evaluated following the WHO standard outlined in the international pharmacopoeia, as detailed in our previous studies [1] [2]. Briefly, materials were suspended at a concentration of 1 mg/mL, and 1 mL of each suspension was combined with 10 mL of fluid thioglycolate medium at a pH range of 6.9–7.3. The solutions were incubated at 37 ˚C for 14 days and inspected daily for signs of bacterial development. During the 14 day incubation period, each sample and control was periodically assessed for the presence of bacterial and fungal colonies utilizing the pour plate method with potato dextrose agar (PDA) and plate count agar (PCA).

Cell culture

Human THP-1 monocytes were acquired from ATCC (Manassas, VA) and were grown in RPMI 1640 medium supplemented with 1% Amphotericin B, 100 U/mL penicillin, 100 μg/mL streptomycin (Life Technologies, Inc., Carlsbad, CA), 10% heat-inactivated fetal bovine serum (FBS), and 10 mM HEPES (Corning, Inc.). THP-1 monocytes were differentiated using the modified phorbol 12-myristate-13-acetate (PMA) (Life Technologies, Inc., Carlsbad, CA) technique suggested by Daigneault et al. [3]. This approach has been demonstrated to generate macrophages that closely mimic primary human macrophages in terms of morphology, phenotypic markers, and function. Briefly, cells were suspended at a concentration of 3.2×10^5/mL in RPMI + 10% FBS containing 200 nM PMA and then plated into 96-well black-walled imaging plates (BD, Franklin Lakes, NJ) at 8.0×10^4 cells (250 μL) per well. Plates were incubated for 3 days at 37°C and 5% CO_2, washed with 250 μL/well of phosphate buffered saline (PBS) (Corning, Inc.), and incubated for an additional 4 days in RPMI + 10% FBS solution without PMA.

Supplementary Figures

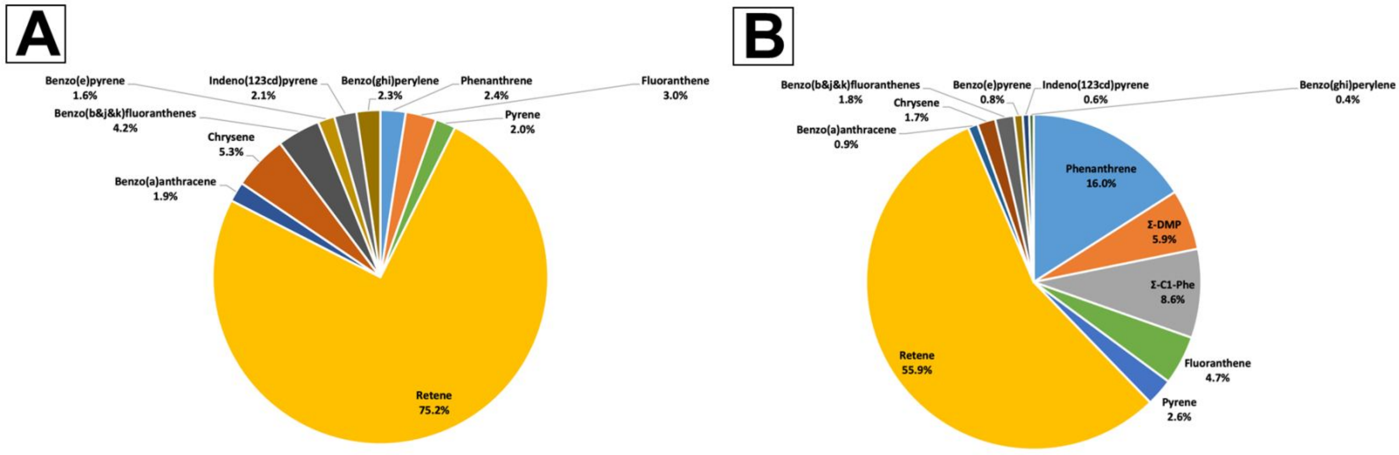

Figure S1. Mass concentration of PAHs in WFPM fractions. **A.** Mass concentration of PAHs in $WFPM_{0.1}$ **B.** Mass concentration of PAHs in $WFPM_{0.1\text{-}2.5}$. Reproduced from [4].Copyright [2024] American Chemical Society.

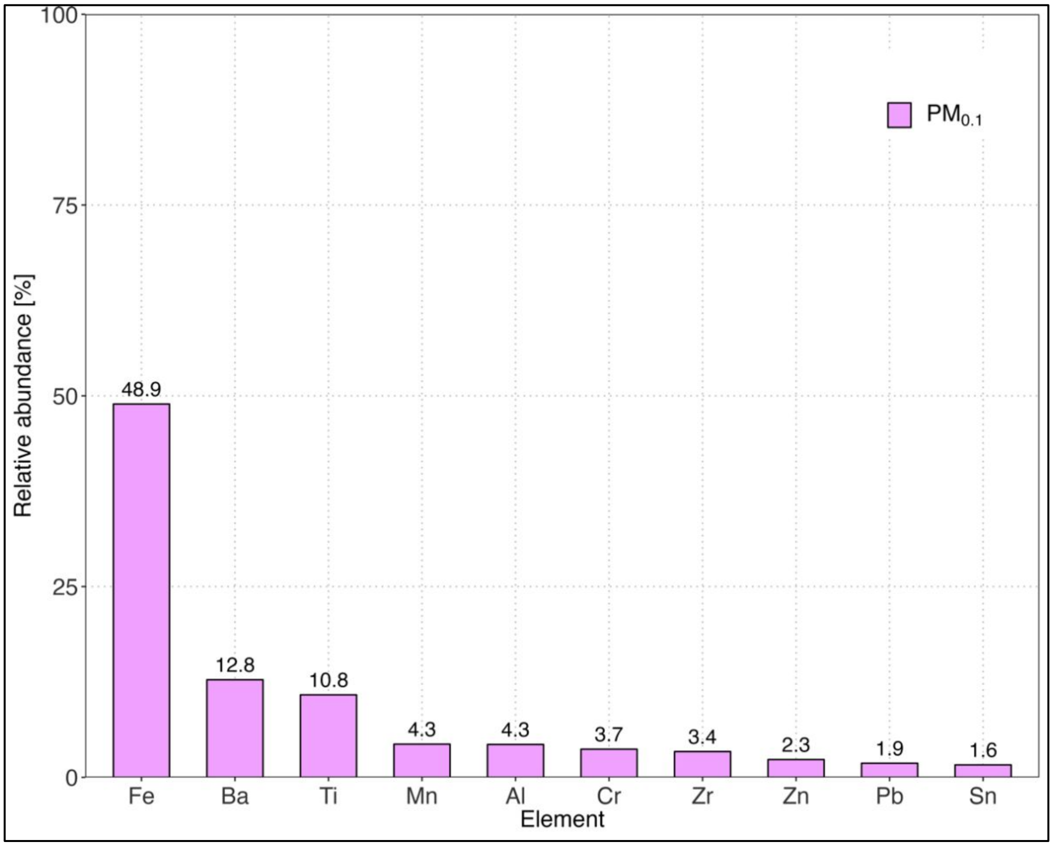

Figure S2. Relative abundance of the ten most prevalent elements in $WFPM_{0.1}$. Reproduced from [4]. Copyright [2024] American Chemical Society.

Supplementary Tables

Table S1. MPPD input parameters. Aerosol mass concentration, Mass Median Aerodynamic Diameter, MMAD, and effective density, ρ_{eff}, used as input parameters for lung deposition calculations by MPPD software.[1]

Particle size fraction	Mass concentration µg/m³	Mass Median Aerodynamic Diameter, MMAD µm	Effective density, ρ_{eff} g/cm³
**$WFPM_{0.1}$**	**165**	**0.05**	**0.955**
**$WFPM_{0.1\text{-}2.5}$**	**165**	**1.30**	**0.480**

[1] Note: The effective density was calculated according to the data measured by Leskinen et al. [5]
Additional MPPD input parameters were outlined by Lizonova et al. using the Yeh/Schum symmetric model (Schum G Yeh H-C, 1980), with a functional residual capacity of 3,300 mL and a head volume of 50 mL [6]. The nasal respiratory rate was set to 12 breaths per minute, the tidal volume to 625 mL, and the inspiratory fraction to 0.5 [7]. The Geometric Standard Deviation (GSD) was set equal to 1 in the model for both WFPM fractions, representing a uniform particle size distribution.

Table S2. Colloidal characterization of WFPM in water and cell culture medium at DSE_{cr} (hydrodynamic diameter d_H, polydispersity index PdI, zeta potential ζ, specific conductance σ and mean effective density $\boldsymbol{\rho}_{EV}$).

	Time (h)	Dispersion	Intensity weighted d_H (nm)	PdI	ζ (mV)	σ (mS/cm)	$\boldsymbol{\rho}_{EV}$ (g/cm^3)
$WFPM_{0.1}$	0	DI WATER	242.9 ± 71.5	0.081 ± 0.017	-34.63± 1.43	0.23 ± 0.004	1.565
	0	RPMI + 10% FBS	263.9 ± 57.8	0.163 ± 0.015	-8.70 ± 0.63	12.9 ± 0.76	
	24	RPMI + 10% FBS	394.4 ± 155.8	0.400 ± 0.082			
$WFPM_{0.1-2.5}$	0	DI WATER	532.4 ± 43.84	0.497± 0.092	-32.3 ± 1.10	0.226 ± 0.003	1.650
	0	RPMI + 10% FBS	474.7 ± 49.06	0.797 ± 0.098	-9.06±1.05	12.9 ± 1.08	
	24	RPMI + 10% FBS	330.2 ± 115.3	0.633 ± 0.158			

1. Pyrgiotakis, G., Luu, W., Zhang, Z., Vaze, N., DeLoid, G., Rubio, L., Graham, W.A.C., Bell, D.C., Bousfield, D., Demokritou, P.: Development of high throughput, high precision synthesis platforms and characterization methodologies for toxicological studies of nanocellulose. Cellulose (Lond). 25, 2303–2319 (2018)

2. Beltran-Huarac, J., Zhang, Z., Pyrgiotakis, G., DeLoid, G., Vaze, N., Demokritou, P.: Development of reference metal and metal oxide engineered nanomaterials for nanotoxicology research using high throughput and precision flame spray synthesis approaches. NanoImpact. 10, 26–37 (2018). https://doi.org/10.1016/j.impact.2017.11.007

3. Daigneault, M., Preston, J.A., Marriott, H.M., Whyte, M.K.B., Dockrell, D.H.: The Identification of Markers of Macrophage Differentiation in PMA-Stimulated THP-1 Cells and Monocyte-Derived Macrophages. PLoS One. 5, e8668 (2010). https://doi.org/10.1371/journal.pone.0008668

4. José G. Cedeño Laurent, Hooman Parhizkar, Leonardo Calderon: Physicochemical characterization of the particulate matter in New Jersey/New York City area, resulting from the Canadian Quebec wildfires in June 2023. Environmental Science & Technology . (2024)

5. Leskinen, J., Ihalainen, M., Torvela, T., Kortelainen, M., Lamberg, H., Tiitta, P., Jakobi, G., Grigonyte, J., Joutsensaari, J., Sippula, O., Tissari, J., Virtanen, A., Zimmermann, R., Jokiniemi, J.: Effective Density and Morphology of Particles Emitted from Small-Scale Combustion of Various Wood Fuels. Environ Sci Technol. 48, 13298–13306 (2014). https://doi.org/10.1021/es502214a

6. Lizonova, D., Nagarkar, A., Demokritou, P., Kelesidis, G.A.: Effective density of inhaled environmental and engineered nanoparticles and its impact on the lung deposition and dosimetry. Part Fibre Toxicol. 21, 7 (2024). https://doi.org/10.1186/s12989-024-00567-9

7. Martin, J., Bello, D., Bunker, K., Shafer, M., Christiani, D., Woskie, S., Demokritou, P.: Occupational exposure to nanoparticles at commercial photocopy centers. J Hazard Mater. 298, 351–360 (2015). https://doi.org/10.1016/j.jhazmat.2015.06.021
